# Double Networks of Liquid-Crystalline Elastomers with Enhanced Mechanical Strength

**DOI:** 10.1021/acs.macromol.1c02065

**Published:** 2022-01-28

**Authors:** Xueyan Lin, Weike Zou, Eugene M. Terentjev

**Affiliations:** † Cavendish Laboratory, University of Cambridge, JJ Thomson Avenue, Cambridge CB3 0HE, U.K.; ‡ State Key Laboratory of Chemical Engineering, Zhejiang University, Hangzhou 310027, P.R. China

## Abstract

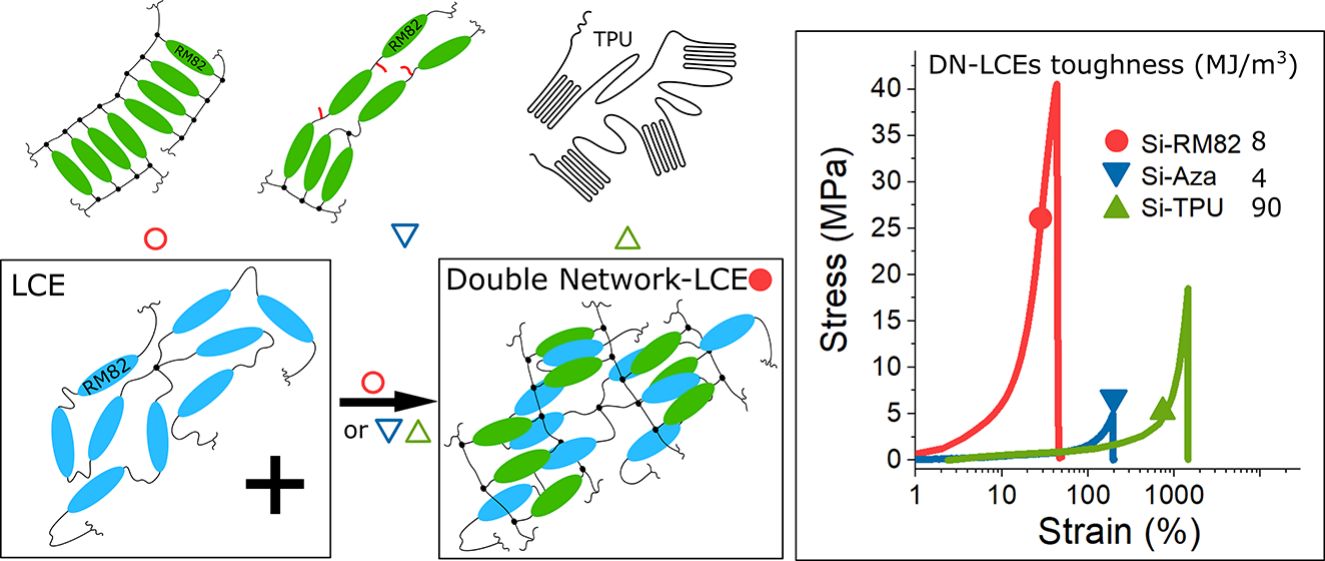

Liquid-crystalline elastomers (LCEs) are frequently used in soft actuator development. However, applications are limited because LCEs are prone to mechanical failure when subjected to heavy loads and high temperatures during the working cycle. A mechanically tough LCE system offers larger work capacity and lower failure rate for the actuators. Herein, we adopt the double-network strategy, starting with a siloxane-based exchangeable LCE and developing a series of double-network liquid-crystalline elastomers (DN-LCEs) that are mechanically tougher than the initial elastomer. We incorporate diacrylate reacting monomers to fabricate DN-LCEs, some of which have the breaking stress of 40 MPa. We incorporate thermoplastic polyurethane to fabricate a DN-LCE, achieving an enormous ductility of 90 MJ/m^3^. We have also attempted to utilize the aza-Michael chemistry to make a DN-LCE that retains high plasticity because of several bond-exchange mechanisms; however, it failed to produce a stable reprocessable LCE system using conventional ester-based reactive mesogens. Each of these DN-LCEs exhibits unique features and characteristics, which are compared and discussed.

## Introduction

Liquid-crystalline elastomers (LCEs) are a unique class of soft materials that incorporate mobile liquid-crystalline ordering with rubber–elastic solids. The balance between the two effects enables LCEs to achieve large, reversible length change on the macroscopic scale when their underlying order parameters change.^1^ Because of their increasingly easy fabrication methods, in recent years, a growing variety of LCE designs were made for use as mechanical actuators.^2−7^ When LCEs are heated, the order parameter decreases and aligned LCEs would contract along with their average mesogen orientation. In this way, LCE actuators convert the energy stored in their phase ordering into mechanical work against the attached load. Because of its simple execution, thermal stimulus is the most common method to actuate contemporary LCEs.^8,9^ However, thermal actuation presents a salient challenge to LCEs’ performance as actuators in practical settings: the elastic modulus of LCEs always decreases as the surrounding temperature increases. This effect becomes more pronounced near the nematic–isotropic transition point when a sudden drop of elastic modulus is observed, often to values much lower than 1 MPa before raising again to the isotropic rubber plateau.^10−12^ Such a softening makes LCEs particular vulnerable to heavy load at high temperatures when the work output is observed to decrease and samples could fail as a consequence.^13^ Therefore, this is a serious problem that obstructs LCEs from becoming practically useful high-stroke actuators operating through continuous heating cycles, in comparison to other types of actuators.^14−16^

To withstand heavy loads and fully exploit the LCEs’ actuation potential, a new strategy to toughen LCEs without losing their actuation capability is needed. In the past, toughening LCEs at ambient temperature has mostly been attempted by introducing additional physical cross-linking, such as additional crystallinity or hydrogen bonding.^11,17−21^ However, at high temperatures these effects are greatly diminished because crystallites melt away and the hydrogen bonding depletes. As a result, these LCEs do not show sufficient toughness at the height of thermal cycles. Furthermore, because the formation of crystallites in LCEs often requires minutes at ambient temperature, the crystallinity method can be used only when repeated thermal actuation is not required. As for hydrogen bonding, evidence suggests that too much of hydrogen bonding often conflicts with mesogen interaction and can result in the disruption of alignment, leading to a loss of actuation reversibility.^22,23^

In this paper, we use the strategy of double networks that offers an alternative solution to the toughening of LCEs. This strategy has been successfully demonstrated in regular hydrogels and elastomers to increase their elastic moduli, breaking stress, and strain-at-breaks, and hence the overall toughness.^24−26^ Generally, the double network is constructed by incorporating a rigid network into a soft base network. The rigid secondary network could be sacrificial during excessive loading, and the additional mechanical energy can be dissipated, resulting in much higher stress to break the sample. The base elastic network serves a different role in preventing the crack propagation and preserving the sample integrity even after the local partial breakdown of the secondary rigid network, so the strain-at-break does not significantly decrease. Consequently, the toughness of the double network increases. In practice, the classical way to fabricate double networks is sequential fabrication where the monomers of the rigid networks are introduced into the base network by absorption and later polymerize *in situ*.^27^ After the formation of double networks, the strength and toughness of the base component are greatly enhanced. Unlike physical bondings, the properties of double networks are less affected by temperature fluctuation because of the stability of covalent bonds in both networks. To date, the majority of double-network research focuses heavily on hydrogels, with only a handful of research groups applying this fabrication method to liquid-crystalline polymer systems to introduce shape-memory functionality as well as enhanced pliancy.^29−31^ Unfortunately, no toughening effect was reported because this strategy works only after a careful design of the ratio between each of the two networks.^27^ A recent paper described a new method of one-pot synthesis of a double-network LCE (DN-LCE) using polyacrylate LCN within a polyurethane LCE.^32^ Their results show a DN-LCE with a remarkable 50 MPa breaking stress measured at room temperature and an 8 MPa breaking stress measured at 140 °C. It is worth noting that in line with our earlier argument on hydrogen bonding disrupting mesogen interaction, the polyurethane LCE they used did not achieve a two-way reversible actuation.

For the practical use of LCEs, their ability to be reprocessed and their mesogen orientation to be reprogrammed is an attractive additional proposition, because the LCEs can therefore be recycled and remanufactured into more complex geometries without losing actuation capability. However, in double-network systems, the introduction of reprocessability has been more challenging. Although the two networks are entangled with each other, they should not covalently cross-link before and after the reprocessing. Initially, we attempted to construct such an orthogonal system with siloxane/transesterification,^41−,37,^ by using a combination of thiol–ene, aza-Michael, and photopolymerization reactions. However, we later realized that the aza-Michale network is participating in side reactions, which results in fusion of the two networks and a decrease of the mechanical strength. We discuss this type of material to highlight its properties, because using the aza-Michael reaction has recently been one of the common ways to fabricate LCEs.^19,21,45^

Herein, we report the fabrication of three different types of DN-LCEs and discuss the effects of the different additional networks on their final properties. First, in all cases, a soft xLCE is used as the base network, which contains siloxane-exchange functionality for network reconfiguration. Using different fabrication methods, this base xLCE is then interpenetrated with three different materials: (1) mesogenic permanent enhancing network and (2) thermoplastic nonmesogenic network. We also explore (3) an “unsuccessful” double network obtained using aza-Michael chemistry. We use the classical method of immersion, where the monomers are diffused into the premade base network before they are radically cross-linked into the enhancing networks. We also explore a one-pot fabrication method where the thermal plastic polymer is diffused during the fabrication of the base xLCE. We then proceed to investigate the resultant three DN-LCEs and compare them in terms of their mechanical properties, their ability to flow at elevated temperatures, and their capacity for thermal actuation.

## Experimental Details

Acrylate mesogenic reacting monomer (RM82, 95% purity) was purchased from Daken Chemical Ltd. Thermoplastic polyurethane filament TPU95A was purchased from Ultimaker. Nonmesogenic monomer tri(propylene glycol) diacrylate (diA) and nonmesogenic monomer bisphenol A diglycidyl ether diacrylate (diBP) are used in control experiments. Chain-extender 3-amino-1,2-propanediol (APD, 97% purity), thiol spacer 2,2′-(ethylenedioxy) diethanethiol (EDDT, 95% purity), siloxane spacer 1,3-divinyl tetramethyl disiloxane (DVS, 97% purity), siloxane cross-linker 2,4,6,8-tetramethyl 2,4,6,8tetravinyl cyclotetrasiloxane (TVCS, 95% purity), thiol–ene catalyst dipropylamine (DPA, 99% purity), siloxane-exchange catalyst 4-(dimethylamino) pyridine (DMAP, 99% purity), photoinitiator Irgacure 651 (I-651, 99% purity), and antioxidant butylated hydroxytoluene (BHT, 99% purity) were all purchased from Sigma-Aldrich and used without purification. We use UV LEDs purchased from New Energy (365 nm, 875 mW) for the photopolymerization.

### Synthesis of the Single-Network Si-SN and Aza-SN

**Figure 1:**
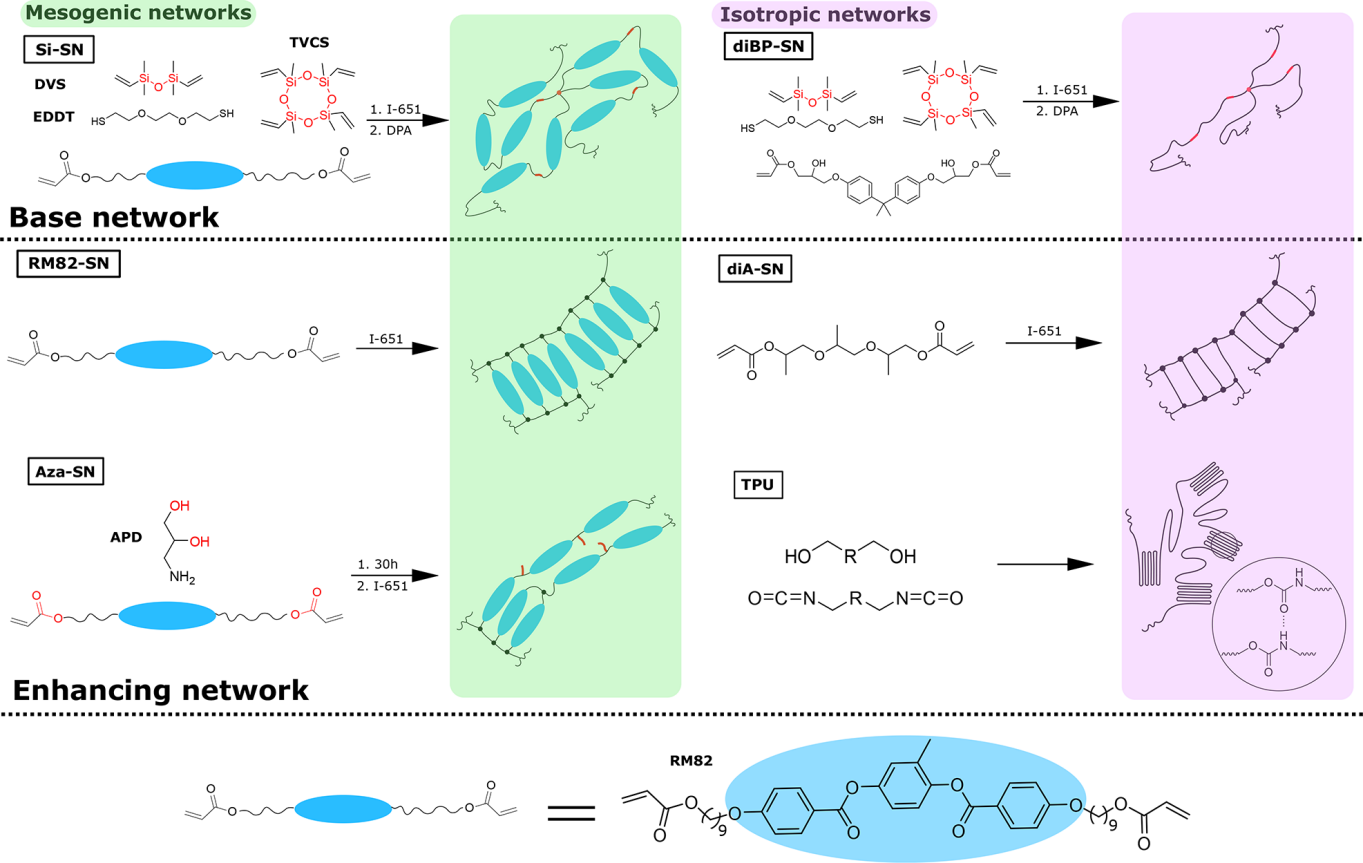
Reaction schemes of each type of single network. These single networks are divided into mesogenic and isotropic, as indicated by green and purple shading. Si-SN is used as the base network, and it is combined with the four types of secondary “enhancing” networks (RM82, Aza, diA, and TPU) into DN-LCEs. On one occasion, diBP-SN was used to replace Si-SN for a control experiment. The functional groups, which are responsible for siloxane exchange and transesterification, are highlighted in the schemes.

A number of single networks were used in this study, and their structures are shown in Figure 1. There were in total six single networks, and they are divided into mesogenic or isotropic categories. Siloxane exchange and transesterification are the two bond-exchange reactions used for making reprocessable DN-LCE, and their enabling functional groups are highlighted in the schemes in Figure 1. In order to fabricate DN-LCEs, Si-SN was used as the fixed base network, combined with different types of enhancing secondary networks (except on one occasion the Si-SN was replaced by the nonmesogenic counterpart diBP-SN for a control experiment). We now describe the steps to prepare stand-alone Si-SN and Aza-SN, because the rest of the single networks are made only during the fabrication of DN-LCEs, which will be discussed later.

#### Si-SN

Si-SN is made by following a procedure used in making siloxane-based xLCE.^41^ RM82 (3.38 g), EDDT (1.26 g), DVS (0.188 g), and TVCS (0.156 g) are used to make multiple Si-SN samples at once in one batch. The synthesis of Si-SN includes two steps: first load EDDT, DVS, and TVCS in a vial along with 10 mg (1 wt %) of I-651. No solvent is added at this step. Stir the liquid mixture while exposing it to 365 nm UV light for 5 min to produce the thiol-terminated oligomer. The resultant viscous oligomer is then combined with RM82 (dissolved in 3 g of toluene), 20 mg (2 wt %) of DPA, and 10 mg (1 wt %) of DMAP (dissolved in a minimal amount of acetonitrile), which is the siloxane-exchange catalyst. Degas the mixture in a vacuum, pour the solution into a homemade PTFE dogbone mold designed for optimal mechanical testing, and cure in a hot press at 60 °C. The samples are then peeled off from the mold and dried in a vacuum overnight.

Initially, dimethylformamide (DMF) was used as the solvent for the synthesis of Si-SN, in order to facilitate the thiol–acrylate reaction speed. However, we found that DMF can react with siloxane-based cross-linker DVCS, so the resultant Si-SN had a lower gel fraction: gel fraction of Si-SN is 78% when using pure DMF as the solvent, but it rises to 85% when using toluene:DMF = 1:1 and to 94% when using pure toluene. Subsequently, we fabricated all Si-SN in toluene. It may appear sensible to replace exchangeable DVCS with a permanent vinyl-based cross-linker, such as (1,3,5-triallyl-1,3,5-triazine-2,4,6(1H,3H,5H)-trione, as it would provide more stability during the fabrication process. But this eliminates the possibility for the base network to flow at high temperatures, which is essential to allow the base network to adapt to the deforming secondary network during alignment.

#### Aza-SN

RM82 (812 mg) and APD (100 mg) are used to make this type of single network through the aza-Michale reaction, with acrylate groups in 10% excess.^13^ Over time, acrylate slowly reacts with the primary amine of APD and generates the acrylate-terminated liquid-crystalline linear polymer, which is then cross-linked under UV by acrylate photopolymerization. First, mix both reagents with 5 mg (0.5 wt %) of BHT, 10 mg (1 wt %) of DMAP, and 360 mg of DMF in a vial. Seal the vial in a nitrogen atmosphere and place it at 90 °C for the next 30 h. The brown-yellow viscous resin containing liquid-crystalline polymer is diluted with an extra 2 g of DMF, and 20 mg of I-651 is added. Degas the resin in vacuum before pouring it into a PTFE dogbone mold and curing under 365 nm UV. The samples are then collected from the mold and dried in a vacuum overnight.

### Synthesis of DN-LCEs

In order to make double networks, Si-SN is used as the base network. Four DN-LCEs were fabricated in this way, with two of them having mesogenic secondary network and two having isotropic secondary networks. These four DN-LCEs, along with an additional one made using the nonmesogenic diBP-SN, are listed in Table 1. For example, Si-RM82 is made by combining the base Si-SN with mesogenic RM82-SN, according to the table, and it is made by the immersion method. The three resulting DN-LCEs that we mainly focused on in this paper are highlighted in bold in Table 1, while the remaining two DN-LCEs were used for control experiments.

**Table 1:** Table of DN-LCEs Used in This Work^a^

DN-LCEs	base SN	enhancing SN	fabrication methods	flow at high *T*	actuation
**Si-RM82**	Si-SN	RM82-SN	immersion	×	√
**Si-Aza**	Si-SN	Aza-SN	immersion	√	√
Si-diA	Si-SN	diA-SN	immersion	×	×
**Si-TPU**	Si-SN	TPU	one-pot	√	√
diBP-TPU	diBP-SN	TPU	one-pot	√	×

a Each sample’s constituent single networks, fabrication methods, ability to plastically flow, and ability to actuate are provided.

#### Si-RM82

RM82 (0.6 g) and 30 mg (2 wt %) of I-651 are dissolved in 2.4 g of toluene under mild heating, making up a clear solution of 20 wt % RM82 concentration. Si-SN dogbone samples synthesized previously are submerged into the solution and allowed to be fully swollen at 60 °C for the next 3 h. The swollen gels are then dried in vacuum to afford the precured Si-RM82 that still contains the absorbed, un-cross-linked RM82 monomers inside. The samples taken out from the oven can be photopolymerized at room temperature into (a) polydomain DN-LCE in a load-free state or (b) aligned monodomain after 200% strain is maintained during UV exposure. For the synthesis of Si-diA, Si-SN is similarly swollen into a 20 wt % nonmesogenic diA solution in toluene then dried in a vacuum oven and photo-cross-linked afterward. After extensive testing, 20 wt % solution was found to be our optimal RM82 concentration in the making of Si-RM82 because the resultant Si-RM82 sample was able to show greater toughness without losing its ability to thermally actuate. The percentage weight increase of Si-SN after monomer absorption is shown in Table 2. The amount of absorbed monomers in this process is 35 wt % to the base Si-SN mass.

**Table 2:** Percentage Weight Increase of Si-SN after Immersing in Different RM82 Toluene Solutions for 3 h at 60°C and Drying^a^

RM82 concentration in solution (wt %)	5	10	20	30
weight increase compared to Si-SN (%)	4	22	35	46

a From a range of tested solutions, we used 20 wt % solution in the rest of this work because it affords Si-RM82 with the optimal toughness/actuation combination.

**Figure 2:**
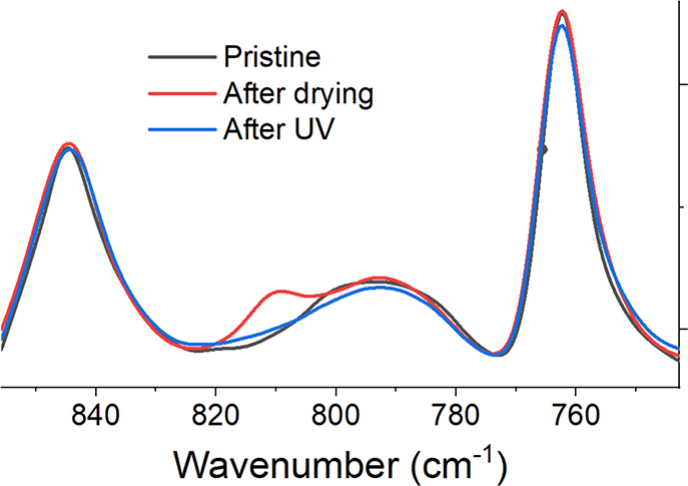
ATR-FTIR shows the acrylate peak (812 cm^–1^) appears after the Si-SN absorbs RM82 monomers from solution. This peak subsequently disappears after UV exposure. The “Pristine” spectrum refers to the initial Si-SN network where there are no unreacted acrylates.

To verify the complete independent formation of the two networks in Si-RM82, an ATR-FTIR test was conducted before and after the Si-SN was swollen in RM82 solution, as well as after the polymerization under UV (see Figure 2).

#### Si-TPU

The amount of TPU polymer added into the network is 50 wt % of the Si-SN amount. Mix 5 mg of I-651, 252 mg of EDDT, 38 mg of DVS, and 31 mg of TVCS in a vial without addition of solvent. Place the vial under UV illumination and photopolymerize EDDT with DVS and TVCS, the same as previously described in the synthesis of Si-SN. In a separate vial add 500 mg of thermoplastic TPU95A and 672 mg of RM82 and dissolve them with 5 g of DMF at 60 °C. When the TPU95A and RM82 fully dissolve, mix the two vials together and add in 50 mg of DPA. Pour the solution into a PTFE mold and leave the mold at room temperature until gelation before evaporating the solvent at 60 °C overnight. For the control isotropic DN-LCE diBP-TPU, repeat the same procedure, but replace the RM82 with an equal molar amount of nonmesogenic monomer diBP.

#### Si-Aza

Prepare 0.5 g of RM82, 61 mg of APD, 5 mg (1 wt %) of DMAP and 10 mg (2 wt %) of I-651. Add in 2.3 g of 10:1 toluene/acetonitrile solvent and dissolve all the reagents at 60 °C. A small amount of acetonitrile is added here to help APD dissolve without precipitating RM82 out. Then, the base Si-SN dogbone samples are added into the vial to swell at 60 °C. The vial is left in the oven for the next 30 h. Remove the dogbone samples after the reaction and clean their surface of the residual liquid-crystalline resin before drying them on a PTFE substrate in a vacuum. The weight increase of Si-SN after this process was 31 wt %, representing the total amount of monomers absorbed. The dried dogbones are then cured under a 365 nm UV light at room temperature—again, either directly in a polydomain form or as an aligned monodomain under extension.

### Characterization

The storage and loss moduli of all samples are obtained in a TA DMA850 instrument, in tensile mode at a fixed 1 Hz and a constant temperature ramping rate of 2 °C/min. The same instrument is also used for recording stress relaxation enabled by bond-exchange reactions. For stress relaxation tests, samples are first heated to 230 °C and stretched by fixed 1% tensile strain for Si-Aza and Si-TPU samples and by 0.1% strain for the much more rigid Si-RM82 sample. Recording the stress relaxation over time lets us compare the relaxation curves for different DN-LCEs. The reprocessability test is carried out by cutting the DN-LCE samples and placing them under a 200 °C hydraulic press for 1 h, in order to observe whether the DN-LCE pieces have fused together because of bond-exchange reaction or remained separate in a true thermoset. Uniaxial tensile tests of DN-LCEs are carried out on a universal testing machine (Tinius Olsen 1ST) at a constant strain rate of 0.01/s, with the breaking stress and strain-at-break of each sample are recorded. Thermal actuation and blocking stress test are again carried out in a DMA850 instrument in tensile mode. In order to test natural thermal actuation, a very low 50 KPa stress is applied to keep the sample taut. Strain (or natural length) change during actuation is recorded when the furnace is cycled between 30 and 150 °C at a ramp rate of 5 °C/min. For the thermal actuation at a high load, 1 MPa stress is applied instead. Blocking stress is the stress generated inside the elastomer as it is heated at a fixed strain. In our case, we measure blocking stress when the sample is fixed at a low 0.1% strain (to keep the sample taut) while being heated from 20 to 160 °C at a rate of 5 °C/min. For the iso-stress creep test in the Si-TPU system, the sample is first loaded with a constant 0.5 MPa stress, and then the furnace is heated from room temperature to 180 °C at a rate of 3 °C/min, while the increasing strain (plastic creep) is recorded against temperature. The toughness of all our DN-LCEs is measured by calculating the stored mechanical energy: the area under each of their stress–strain curves until the failure point.

## Results and Discussion

We begin our discussion with the Si-RM82 double network, which combines the exchangeable Si-SN and the permanently cross-linked RM82-SN network. This type of DN-LCE has a high breaking stress and a large elastic modulus. We then proceed to one-pot synthesis of Si-TPU DN-LCE. This type of DN-LCE displays an enormous ductility, with only slightly lower breaking stress compared to Si-RM82. In the end, we explore a combination of Si-SN and Aza-SN. We found this type of DN-LCE is able to relax stress completely by plastic creep at high temperatures. However, it comes at the cost of reduced strength. We also noticed side reactions taking place in Si-Aza at high temperatures, which can result in a permanent lose of mesogenic power and a reduction in the cross-linking density.

### Si-RM82 Double Network

**Figure 3:**
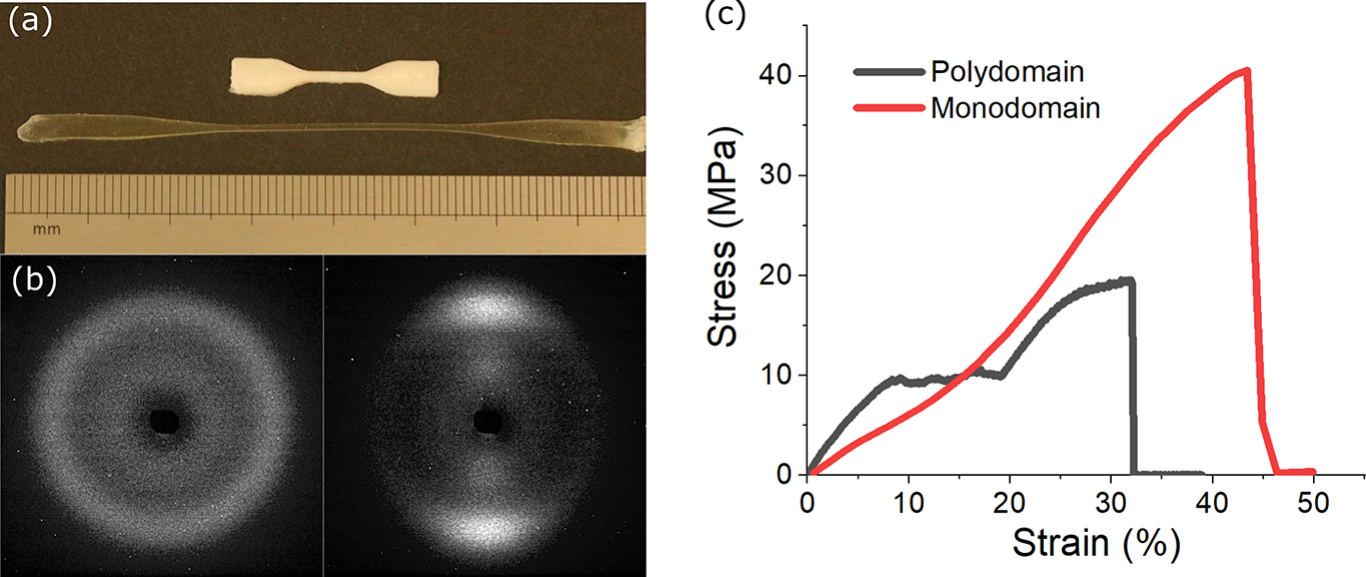
(a) Top: polydomain Si-RM82 sample cross-linked at room temperature without strain imposed, compared to monodomain Si-RM82 (bottom) that is cross-linked when a 200% strain is imposed. (b) The wide-angle X-ray diffraction pattern of polydomain Si-RM82 (left) is compared with the pattern of monodomain Si-RM82 (right), which confirms the mesogen alignment. (c) Both Si-RM82 samples are much stiffer than the base Si-SN (shown later). Polydomain Si-RM82 displays a serrated plateau attributed to the breakdown of the RM82-SN sacrificial network, while monodomain Si-RM82 does not have such a plateau.

Si-RM82 samples are made by swelling the Si-SN base network in toluene containing mesogenic monomers RM82 with photoinitiator. The precured Si-RM82 is opaque after drying because the base Si-SN is in its polydomain state. When a sufficiently large strain is applied, the precured sample becomes transparent indicating the aligned monodomain state. That is, the presence of RM82 monomer does not affect the alignment of Si-SN. In contrast, these monomers can be also aligned by the same mechanical strain because of the template effect of the intact liquid-crystalline order inside the Si-SN.^46^ The overall process is therefore analogous to that found in the “polymer-stabilized liquid crystal” (PSLC) scaffold where mesogens are aligned by liquid-crystalline polymer filaments nearby.^47,48^ Si-RM82 samples fabricated by UV-curing them in an aligned state are found to be permanently fixed in that state. In contrast, if we UV-cure the sample while no strain has been induced, the Si-RM82 remains opaque and fixed permanently in its polydomain state. Figure 3a shows the images of the fabricated opaque polydomain Si-RM82 and the transparent monodomain Si-RM82. The X-ray diffraction measurement confirms that mesogens have been permanently aligned in Si-RM82 (Figure 3b). To demonstrate that the two networks are not chemically linked, fresh Si-SN before and after RM82 absorption, as well as after its UV exposure, are characterized by ATR-FTIR. This result is shown in Figure 2.

This fixed mesogen alignment during the fabrication of Si-RM82 can dramatically affect their uniaxial tensile behavior, and the typical results are shown in Figure 3c. From this graph we notice several features: (1) Without the apparent cost of ductility, the monodomain Si-RM82 has over twice the breaking stress (40 MPa) of polydomain Si-RM82 (18 MPa), due to the anisotropy of RM82-SN therein. A similar relationship between network anisotropy and tensile strength has been previously reported in double-network hydrogel systems, although to a less pronounced degree.^49^ (2) Both Si-RM82 samples display a much stiffer tensile response compared to the base soft Si-SN (shown later), which is again attributed to the rigid RM82-SN. (3) Not seen in monodomain Si-RM82, a serrated plateau existing at around 12 MPa in all the polydomain systems was systematically observed. Such a feature is undoubtedly the consequence of isotropic polyacrylate network being broken down into smaller clusters.^50^ For the same reason, we notice that after this plateau the strain of polydomain Si-RM82 is already very close to sample failure. Such a plateau does not appear in monodomain Si-RM82, which we assume is due to the aligned polyacrylate filaments. In conclusion, cross-linking the precured Si-RM82 in its monodomain state produces Si-RM82 that has a significantly increased breaking stress without apparent cost to ductility.

**Figure 4:**
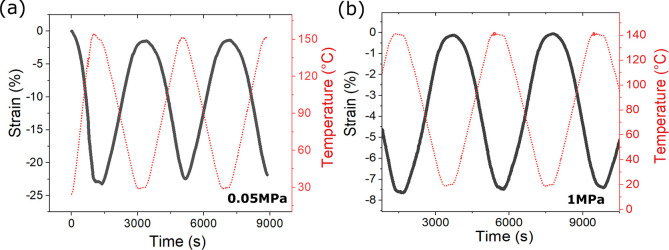
(a) Si-RM82 thermally actuated when under stress-free (50 kPa to keep taut), and (b) when under 1 MPa; heating/cooling rate is 5 °C/min. The samples display actuation across a large temperature range.

**Figure 5:**
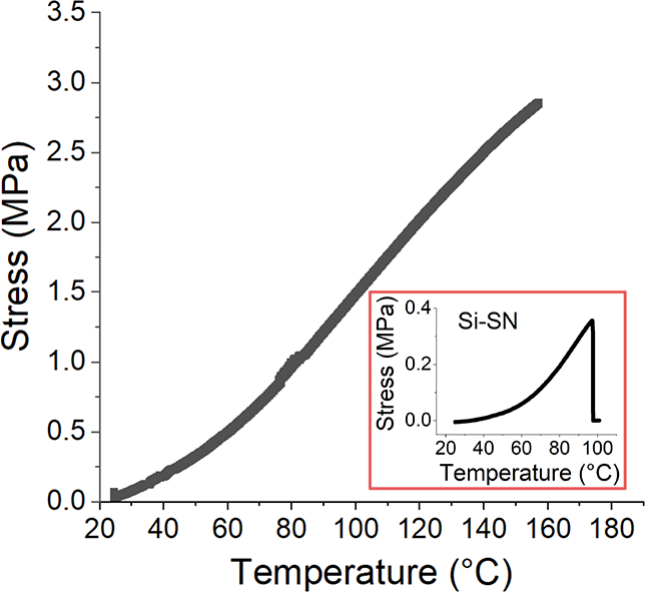
Blocking stress profile (heating rate, 3 °C/min) in Si-RM82 confirms such large actuation temperature range as it has not reached equilibrium at 160 °C. Si-RM82 can sustain a large block stress (3 MPa) without sample failure. In comparison, Si-SN generates 0.35 MPa blocking stress and readily fails at 100 °C (inset).

Monodomain Si-RM82 is strong, but its enhancing RM82-SN network does not completely prevent the base Si-SN from its reversible thermal actuation. This, of course, is dependent on the proportion of polyacrylate in the system, and we can control it by choosing the optimal concentration of RM82 monomer in toluene solution (20 wt %) during the fabrication of Si-RM82. Figure 4 shows the monodomain Si-RM82 sample is capable of actuation under load-free conditions, as well as under large tension. Almost 20% of actuation strain is achievable when the sample actuates stress-free, and 8% of strain remains when the sample actuates under stress as large as 1 MPa. In addition, we notice that the Si-RM82 system actuates over an unusually large temperature range. To verify this observation, we measured the blocking stress generated when the Si-RM82 is heated at a constant length (see Figure 5). The profile spans across a large temperature range, and it is smoother than the blocking stress profile generated in Si-SN (inset). This feature was not reported in the double networks made by Lu et al., and we attribute it to the retained ordering of the RM82-SN network at high temperatures, when the underlaying Si-SN becomes isotropic.^32,46^ Because of the enhanced elastic modulus (shown later), the highest blocking stress generated in Si-RM82 is measured to be 3 MPa, and the sample does not break at this temperature and stress level. This is in stark contrast to the 0.35 MPa in Si-SN which readily breaks down at 100 °C.

Because the rigid RM82-SN network we have used so far is made of reactive mesogen monomers which is an anisotropic molecule, we wanted to know if similar observations can be made if we use an isotropic diacrylate monomer to replace RM82. We therefore synthesized a control network labeled as Si-diA, using isotropic diA-SN (shown in Figure 1) as the absorbed monomer. In this case, the precured Si-diA sample remains transparent after solvent evaporation, indicating the complete loss of liquid-crystalline ordering due to the 20% isotropic impurity added.^51−53^ It responds to imposed strain like any isotropic elastomer, that is, bouncing back upon release of strain. In short, the diacrylate monomer used in this type of DN-LCE to form the rigid enhancing network needs to be mesogenic so that it can be aligned with the base LCE network.

### Si-TPU Double Network

Although logically simple, the immersion method is generally suboptimal: it is time-consuming, requires multiple intervention steps, and may suffer from diffusion limitation in large-sized sample. Thus, a one-pot reaction is favorable as it allows shorter fabrication time and also is scalable. We used a simple one-pot method to fabricate Si-TPU by doping into 50 wt % commercial thermoplastic polyurethane (TPU95A) during the initial Si-SN synthesis. Although the polyurethane used here is not cross-linked, for simplicity we also name this type of sample DN-LCE as they are mechanically not inferior to other DN-LCEs in this report. The resultant Si-TPU is shown to have preserved a liquid-crystalline phase based on the comparison with a control isotropic sample (diBP-TPU), which is made by replacing the base Si-SN network with isotropic diBP-SN.

**Figure 6:**
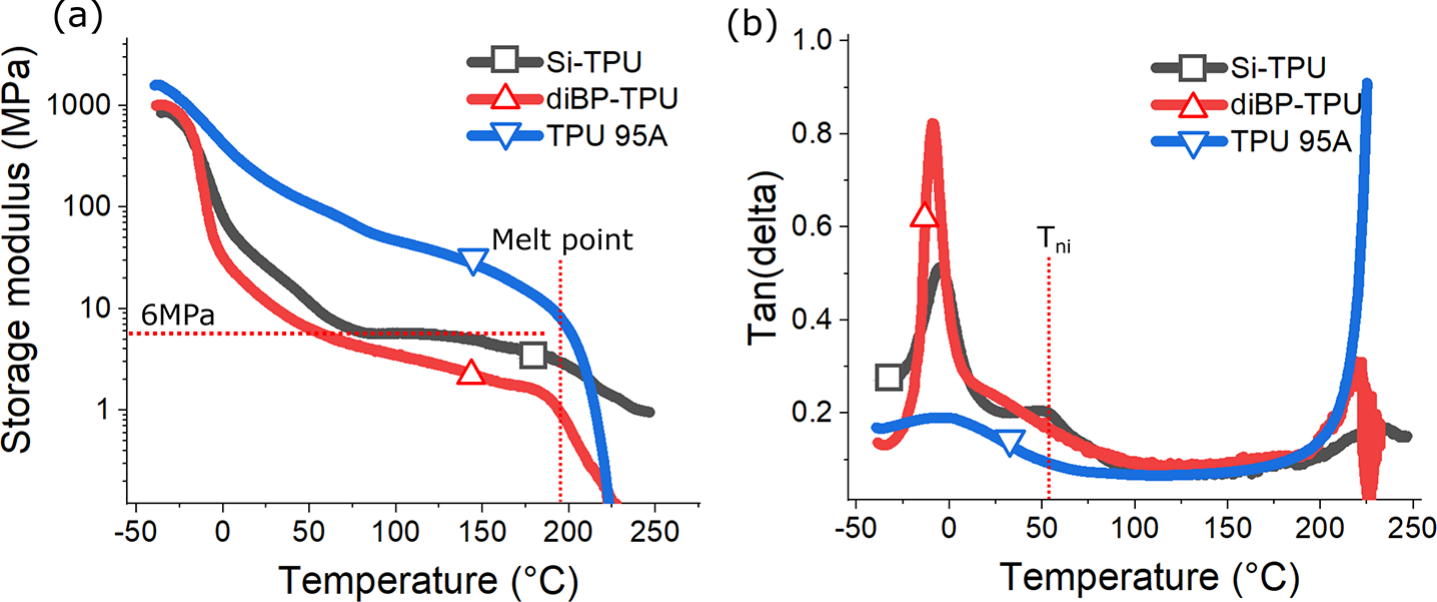
(a) Storage modulus of Si-TPU and diBP-TPU in DMA test, compared to TPU95A. Si-TPU has a relatively stable modulus plateau of 6 MPa after its nematic–isotropic transition at around 50 °C, while diBP-TPU displays a continuous drop of modulus after its glass transition. Both DN-LCEs show a fast decrease of storage modulus at around 200 °C due to the melting of crystallite in their contained TPU.

**Figure 7:**
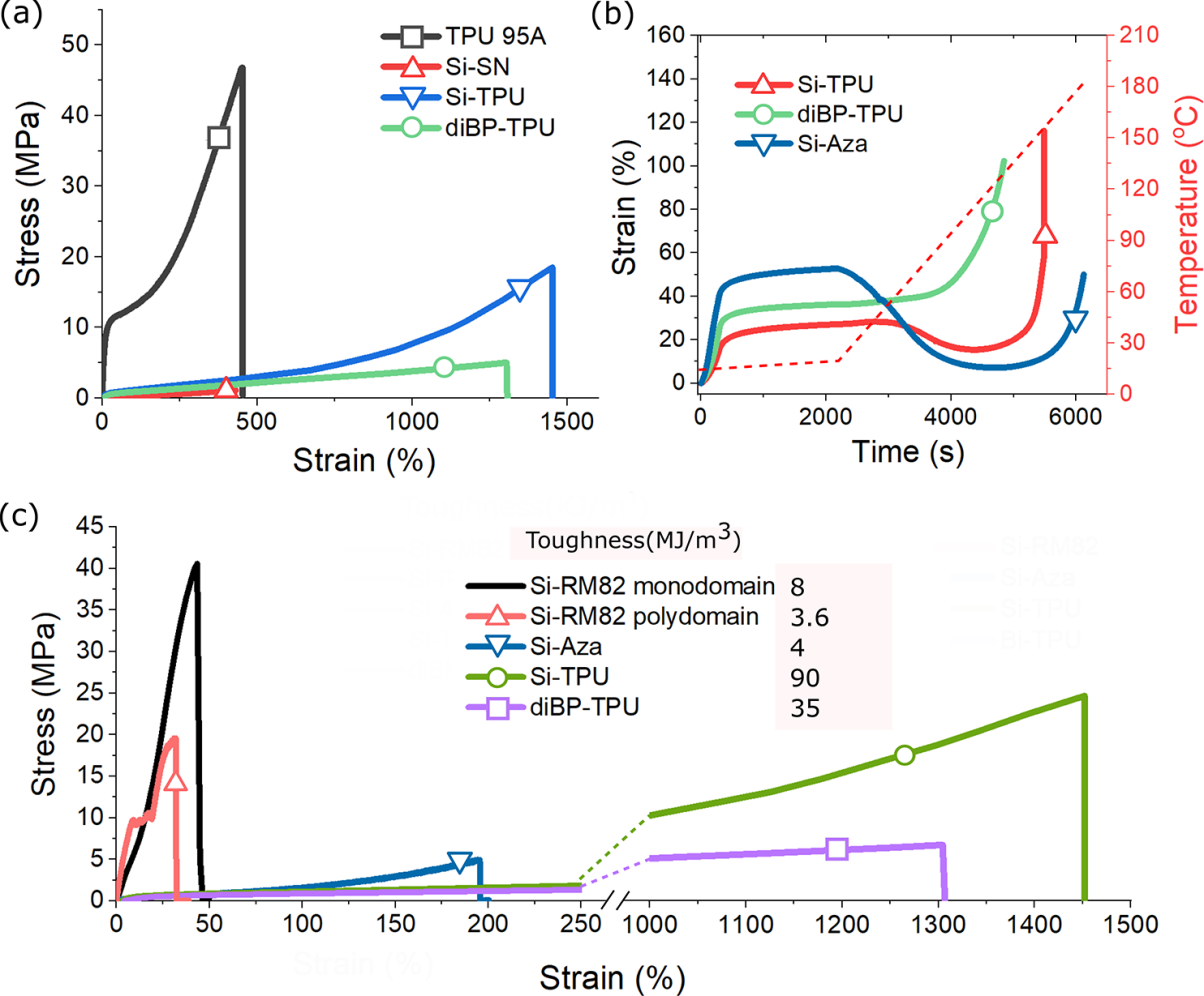
(a) Ambient tensile test of Si-TPU and diBP-TPU in comparison with TPU 95A and Si-SN. Both DN-LCEs show enormous strain-at-break not found from the single networks. (b) Iso-stress test of Si-TPU where it is heated under constant stress of 0.5 MPa. Mesogenic Si-TPU shows a 10% actuation strain while the nonmesogenic diBP-TPU does not actuate at all. For comparison, Si-Aza was also tested under the same heating profile but under a lower 0.25 MPa, showing a similar pattern of behavior. (c) Comparison of the stress–strain curves for Si-RM82 (both poly- and monodomain), Si-Aza, Si-TPU, and diBP-TPU (all polydomain), and each of their toughness values is calculated by measuring the area under curves.

Upon comparison of Si-TPU and diBP-TPU in the DMA test shown in Figure 6a, Si-TPU has a relatively flat rubbery modulus after its nematic–isotropic transition temperature, whereas diBP-TPU shows a continuous drop in modulus. Both samples start to experience a fast drop in storage modulus after the temperature passes 200 °C because of the melting of TPU they contained (TPU 95A). Figure 6b shows the tan(δ) of Si-TPU with an extra peak at around 50 °C, corresponding to nematic–isotropic transition, while diBP-TPU does not have this feature. This suggests that the liquid-crystalline ordering in Si-SN has not been disrupted by the introduction of isotropic TPU. When TPU melts in these DN-LCEs at higher temperatures, the increase of tan(δ) in both DN-LCEs is much less pronounced than pure TPU sample, because the interpenetrating Si-SN has helped protect the network’s mechanical integrity. Remarkably, and perhaps because of the non-cross-linked nature of TPU, both Si-TPU and diBP-TPU display enormous strain-at-break of around 1500%. Tensile test results shown in Figure 7a display such synergy in effect, where the combination of TPU and Si-SN gives rise to significant toughness. The plot in Figure 7c compares their stress–strain curves with those measured in Si-RM82 and Si-Aza (discussed later). If we calculate DN-LCEs toughness by measuring the area below their stress–strain curves, the toughness of Si-TPU is the largest among all fabricated DN-LCEs, reaching 90 MJ/m^3^. It is over 10 times the toughness of Si-RM82 (8MJ/m^3^) and 20 times that of Si-Aza (4MJ/m^3^). This is much more than the toughness of single-network Si-SN and Aza-SN, which are calculated from Figure 10 to be only 2.3 and 3.3 MJ/m^3^, respectively. We note that one expects the toughing effect in Si-TPU to prevail only at room temperature: at high temperatures, the hydrogen bonding will lose its efficacy.

**Figure 8:**
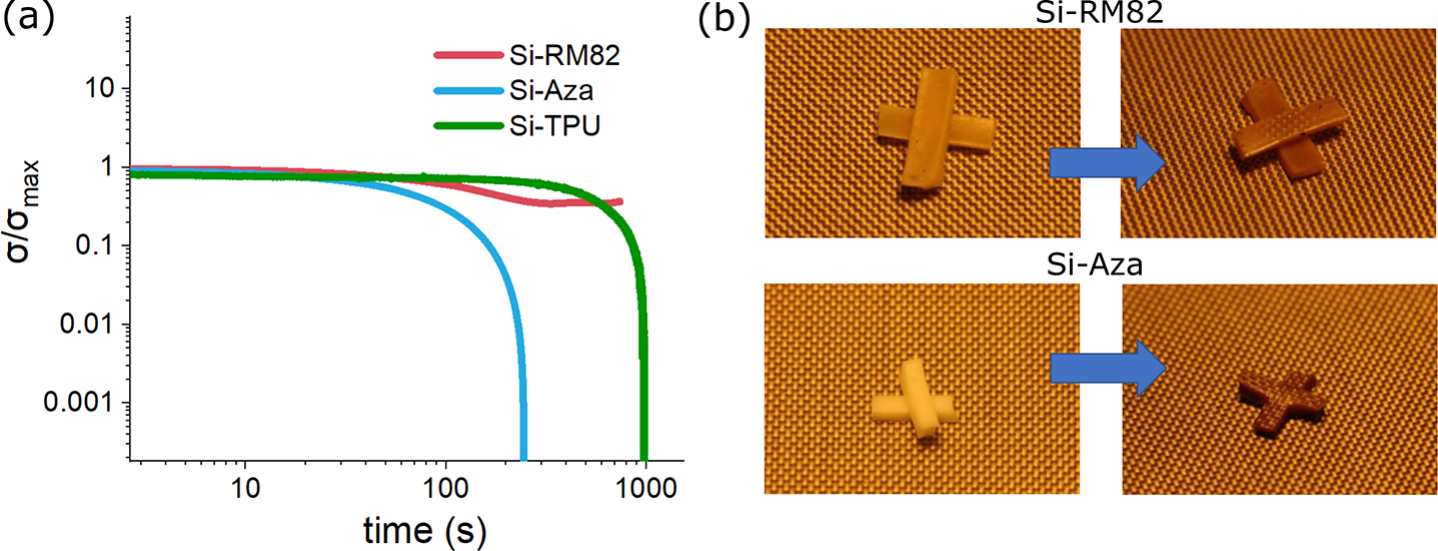
(a) DMA measurement of stress relaxation for the DN-LCEs at 230 °C, where Si-Aza and Si-TPU show a complete stress relaxation, while Si-RM82 does not (the Si-TPU system is introduced in the text). (b) Both DN-LCEs are cut into pieces and pressed under hot press at 200 °C for 1 h. Si-RM82 does not fuse together, while Si-Aza does.

Because we found that liquid-crystalline phase is preserved in the Si-TPU sample, we now want to test whether this type of DN-LCE can thermally actuate. Si-TPU thermal actuation under a constant load is tested by DMA, and the results are shown in Figure 7b. As expected, Si-TPU can thermally actuate under the stress while its isotropic counterpart diBP-TPU does not show any significant contraction at the same stress. Both double networks start flowing at higher temperatures because of the exchange of siloxane and the melting of TPU. For comparison, Si-Aza was also tested in this way, and the result is also included in Figure 7b to show a similar actuation and flowing behavior. However, despite the complete stress relaxation of Si-TPU observed in DMA (Figure 8a), our effort to realign the Si-TPU mesogens at high temperature and program a uniform monodomain have failed, and we could fabricate Si-TPU only in its polydomain state. This is perhaps due to the overwhelming hydrogen bonding that causes constraints in mesogen realignment. In fact, after the addtion of TPU, the Si-TPU responds to imposed mechanical strain just like normal isotropic elastomers do (with a clear lack of semisoft stress plateau), and it remains opaque no matter how much strain was applied. However, if we compare the Si-TPU and Si-diA, both DN-LCEs contain isotropic enhancing networks, but only Si-TPU still preserves liquid-crystalline phase of Si-SN; therefore, it can actuate under load, whereas in Si-diA the original liquid-crystalline phase Si-SN is fully destroyed. Therefore, it is logical to attribute such deviation to the local phase separation in Si-TPU (due to polarity difference of the two constituent networks). Very recently, Gao et al.^54^ described a method to prepare an interpenetrating network using thiol–ene nanogel with linear polyurethane network, which shows the phase separation between the two polymers when the thiol–ene network does not contain a urethane bond. This observation corresponds to our case quite well. In contrast, in Si-diA the isotropic impurity remains homogeneously dispersed and suppresses the mesogenic order.

### Si-Aza Double Network

We attempted to introduce postpolymerization reprocessability in an another type of DN-LCE system, which is labeled as Si-Aza in Figure 1. Instead of RM82 monomers absorbed and photo-cross-linked, here we want a different kind of monomer to be absorbed and photo-cross-linked, whose secondary network has a bond exchangeable mechanism different from siloxane exchange in the base network. We considered that RM82/amine is a suitable monomer combination to be absorbed into the swollen Si-SN because their aza-Michael reaction is slow enough and the monomers have sufficient time to diffuse into the bulk base Si-SN, which is necessary for homogenization.^13,19,57−59^ By a slight off-stoichiometry effect, the growing polymer chains can be capped with acrylate end-groups. In situ generated Aza-SN is thus formed by cross-linking the linear polymers after the evaporation of the solvent. Transesterification is the bond-exchange mechanism in this network, and to facilitate its speed we choose an amine chain-extender APD because of the hydroxyl groups it contains.^60^ Indeed, when combined with siloxane exchange from the base Si-SN, the overall Si-Aza is able to fully relax stress at a test temperature of 230 °C (Figure 8a) in contrast to the Si-RM82 double network. We compared the welding behavior of Si-Aza and Si-RM82 in the hot press, as shown in Figure 8b, clearly confirming the distinction between an exchangeable Si-Aza network and the thermoset Si-RM82.

**Figure 9:**
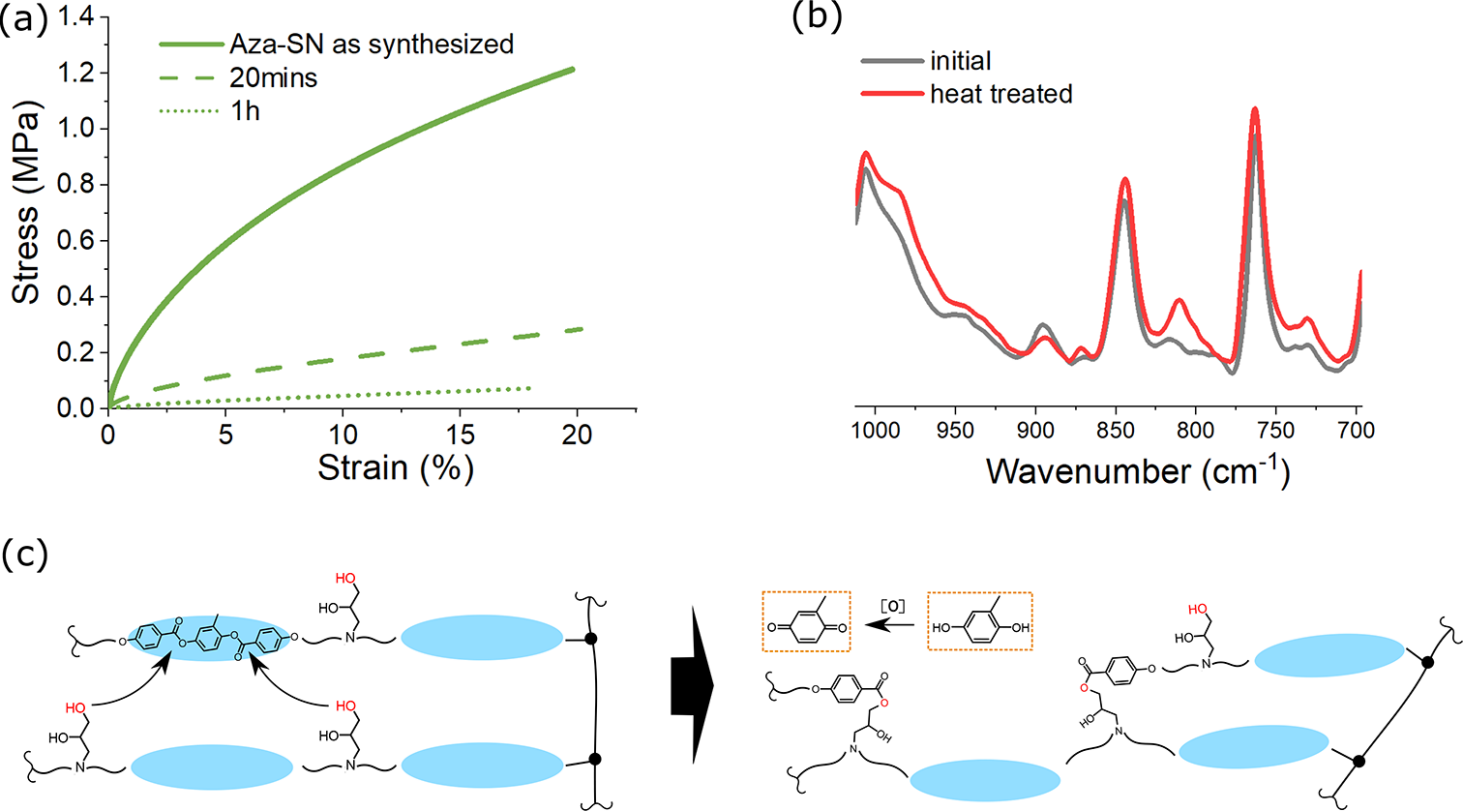
(a) Strain–stress responses of a highly cross-linked Aza-SN, before and after heat treatment at 200 °C for the duration indicated. Each sample is subsequently cooled at room temperature for 24 h prior to the next round of tensile testing. The graph clearly indicates that mechanical deterioration has occurred in the Aza-SN network. (b) Additional peaks appear in the ATR-FTIR scans after the sample was thermally treated, indicating that side reactions have occurred. (c) Illustration of possible excessive transesterification that breaks down the mesogenic core. The newly formed diol is then oxidized by air, producing the brown color.

The plastic flow behavior may be attributed to multiple origins in this network: (1) Independent siloxane exchange and transesterifcation in either of the networks is the ideal scenario that we were hoping to achieve. (2) The additional transesterification across the two networks could occur, because of the additional ester group contained in Si-SN, and thus the two networks can fuse into one. (3) The RM82 mesogen core could break down because of additional transesterification.^38^ (4) A dissociative (retro) aza-Michael reaction could occur within the network, liquidizing it.^61^ In fact, the retro aza-Michael is suggested in our case because we had also tried synthesizing Aza-SN using primary amine that does not contain any hydroxyl group, and that network still can be liquidized at high temperatures (200 °C). Furthermore, we also noticed that the Si-Aza sample turns brown and significantly reduces its tensile strength after heat treatment at around 200 °C, a temperature used for reprocessing (shown in Figures 8b and 9). In order to isolate the process, we fabricated Aza-SN that has acrylate in a high 60% excess, so as to stiffen the sample for tensile measurement. The resulting tensile strain–stress curves, with their corresponding ATR-FITR spectra, were recorded before and after the heat treatment at 200 °C and are shown in Figure 9a,b. The deterioration of mechanical integrity in the sample is obvious after heat treatment as short as 20 min, and the acrylate peaks reappear in its ATR-FTIR spectrum, indicating the retro aza-Michael dissociation.

**Figure 10:**
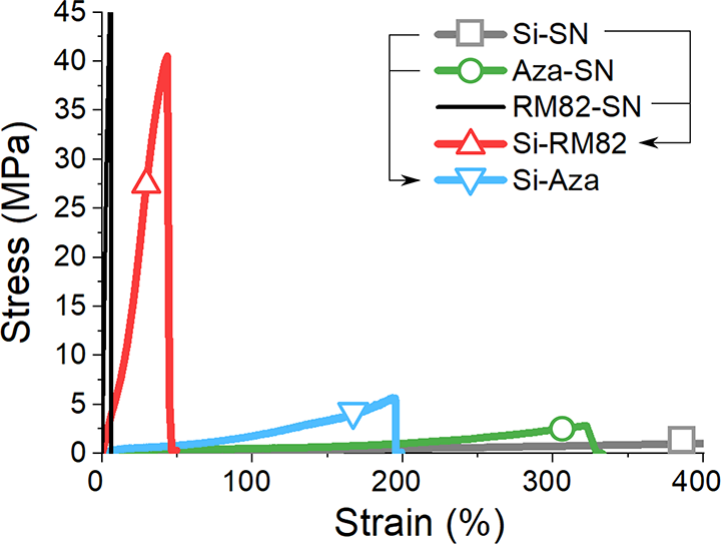
In comparison, Si-RM82 is tougher than its constituent networks: Si-SN and RM82-SN (reproduced from ref (32)). Si-Aza also has a higher breaking stress (5 MPa) than its constituent networks, Si-SN (1 MPa) and Aza-SN (3 MPa), although it is lower than that of Si-RM82. Si-Aza, Si-SN, and Aza-SN samples are all tested in a uniaxial tensile machine in their polydomain state, while monodomain Si-RM82 is used.

**Figure 11:**
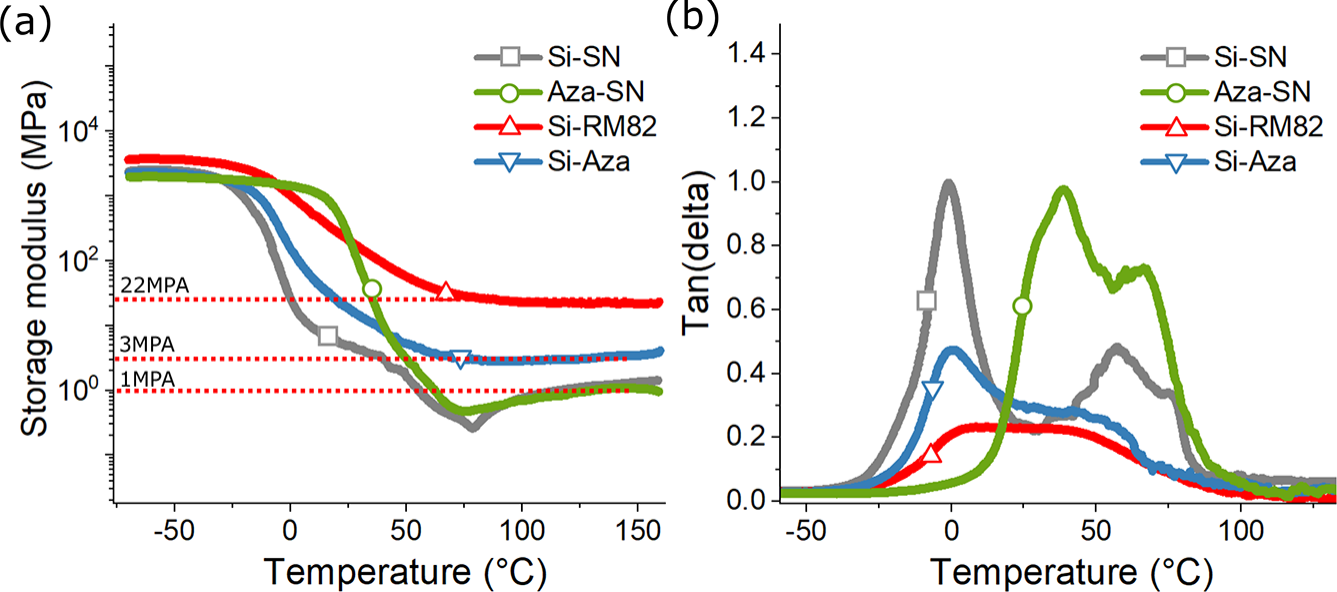
DMA test results of DN-LCEs and single-network Si-SN and Aza-SN. (a) Rubbery modulus is measured in the plateau when samples were heated above 70 °C. Si-RM82 has a modulus of 22 MPa, and Si-Aza has a modulus of 3 MPa; both single networks have a modulus of only 1 MPa. (b) Tan(δ) of the same samples. While all the samples show nematic–isotropic transition temperature at around 70 °C, the transition peaks of DN-LCEs are apparently less obvious than those of single networks.

If we avoid the possibility of degradation in the Si-Aza network by not treating it at high temperatures, the introduction of a low-cross-linking density (soft) Aza-SN network into the soft Si-SN provides an insufficient strengthening effect. In fact, Si-Aza is weaker compared to Si-RM82, as shown in Figure 10. Although because of the presence of a physical sacrificial mechanism (i.e., hydrogen bond), Si-Aza manages to reach 5 MPa breaking stress, slightly better than that of Si-SN (1 MPa) and Aza-SN (3 MPa) alone. The mechanical properties of the four samples can be further revealed by dynamic mechanical analysis (DMA) (shown in Figure 11). In Figure 11a, we can clearly observe variation of isotropic rubbery modulus in different DN-LCEs, with Si-RM82 reaching 22 MPa and Si-Aza 3 MPa. Both DN-LCEs do not show a clear-cut isotropic transition in high temperatures, which is attributed to the internal constraint introduced by the secondary network. In comparison, both Si-SN and Aza-SN have a rubbery modulus of around 1 MPa after seeing a sharp dip in their modulus at around 70 °C, indicating the nematic–isotropic transition. Figure 11b presents the tan(δ) measured from the same samples. We are able to see that all four samples have almost identical nematic–isotropic transition temperatures at around 70 °C, but the nematic–isotropic peaks on both DN-LCEs are much less obvious than those of single networks. Because of the significant presence of hydrogen bonds in Aza-SN, this sample sees an increase in the glass transition temperature compared with the rest of the samples.^62−64^

## Conclusion

In this paper, we have described how to use the double-network strategy to fabricate mechanically tough DN-LCEs that are capable of thermal actuation. We explored three very different DN-LCEs (Si-RM82, Si-TPU, and Si-Aza) obtained by immersion or one-pot synthesis methods. The properties of these DN-LCEs were characterized and compared. For Si-RM82, we found how to fabricate the monodomain sample, which can repeatedly thermally actuate, and compared it with its polydomain counterpart. The monodomain Si-RM82 has the highest breaking stress among all DN-LCEs because of the presence of an aligned rigid polyacrylate network. We showed that this polyacrylate network imparts large rubber modulus to the Si-RM82 and in return increases the blocking stress generated inside Si-RM82 without sample failure. For the thermoplastics double-network Si-TPU, we showed that it has a medium tensile strength and modulus among all our DN-LCEs. Despite the un-cross-linked and isotropic nature of the TPU used in this fabrication, Si-TPU shows a remarkably large strain-at-break; hence, at room temperature, it gives an enormous sample toughness. We also discovered that the Si-TPU is able to thermally actuate under stress, and we discussed the question of why the addition of a nonmesogenic impurity does not adversely affect the liquid-crystalline ordering in the Si-TPU network, as it does in Si-diA. However, the introduction of thermoplastic polyurethane makes this type of DN-LCE not able to be realigned and thus not reprogrammable. For the exchangeable Si-Aza, we fabricated the LCE sample by generating a low-cross-linking-density liquid-crystalline polymer in situ and showed that the sample is able to flow at high temperature and fully relax the externally applied stress. However, the sample sees the lowest tensile strength among all our DN-LCEs because of smaller secondary cross-linking density. We also found the undesired side-reactions that occurred in Si-Aza that cause degradation of mesogens and the network. Through this paper, we demonstrated that the double-network strategy can be employed in making DN-LCEs to improve the mechanical proprieties of LCEs and impart very different characteristics depending on the enhancing network selected.
